# *In situ* effect of a proanthocyanidin mouthrinse on dentin subjected to erosion

**DOI:** 10.1590/1678-7757-2020-0051

**Published:** 2020-10-19

**Authors:** Fabrícia CARDOSO, Ana Paula BOTEON, Tamires Alves Pereira da SILVA, Anuradha PRAKKI, Linda WANG, Heitor Marques HONÓRIO

**Affiliations:** 1 Universidade de São Paulo Faculdade de Odontologia de Bauru Departamento de Dentística, Endodontia e Materiais Odontológicos Bauru Brasil Universidade de São Paulo , Faculdade de Odontologia de Bauru , Departamento de Dentística, Endodontia e Materiais Odontológicos , Bauru , Brasil .; 2 Universidade de São Paulo Faculdade de Odontologia de Bauru Departamento de Odontopediatria, Ortodontia e Saúde Coletiva Bauru Brasil Universidade de São Paulo , Faculdade de Odontologia de Bauru , Departamento de Odontopediatria, Ortodontia e Saúde Coletiva , Bauru , Brasil .; 3 University of Toronto Faculty of Dentistry Department of Clinical Sciences (Restorative) Toronto Canada University of Toronto , Faculty of Dentistry , Department of Clinical Sciences (Restorative) , Toronto , Canada .

**Keywords:** Tooth wear, Dentin, Tooth erosion

## Abstract

Proanthocyanidin has been shown to be efficient in inhibiting matrix metalloproteinases.

**Objective:**

The aim of this *in situ* study was to evaluate the protective effect of Proanthocyanidin-based mouthrinses either with naturally acidic or with a neutral pH applied on dentin subjected to erosion.

**Methodology:**

Eight volunteers wore one palatal device in two phases (7 days washout) with 16 samples per group (n=8). The groups under study were: First Phase/ G1 – 10% proanthocyanidin mouthrinse (pH 7.0, Experimental group 1 – Purified Grape Seeds Oligomeric Proanthocyanidins), G2 – 10% proanthocyanidin mouthrinse (pH 3.0, Experimental group 2 – Purified Grape Seeds Oligomeric Proanthocyanidins). Second Phase/ G3 – 0.12% chlorhexidine mouthrinse (pH 7.0, Positive control group), G4 – no previous treatment (Negative control group). Each device was subjected to 3 erosive cycles (5 minutes) per day for 5 days. Treatments with different mouthrinses were applied once after the second erosive challenge (5 minutes). Profilometry was used to quantify dentin loss (µm).

**Results:**

Data were analyzed by repeated measures of ANOVA followed by Fisher’s test (p<0.05). G1 (1.17±0.69) and G3 (1.22±0.25) showed significantly lower wear values with no statistical difference between them. G2 (2.99±1.15) and G4 (2.29±1.13) presented higher wear values with no significant differences between them.

**Conclusion:**

The 10% proanthocyanidin mouthrinse (pH 7.0) could be a good strategy to reduce dentin wear progression.

## Introduction

Dental erosion is the loss of superficial dental tissue caused by acids of intrinsic or extrinsic origin without the involvement of microorganisms. ^1 , 2^ It can affect both enamel and dentin; however, dentin presents a more complex demineralization process ^4^ due to its higher organic content. ^3^ The organic matrix of dentin is composed of 90% collagen fibrils (type I collagen) and 10% proteoglycan and glycosaminoglycan phosphoproteins. ^3^ The dentin also contains host enzymes such as matrix metalloproteinases (MMPs) and cysteine-cathepsins, which are activated when the pH decreases below 4.5. ^5 , 6^ These enzymes are responsible for the degradation of the demineralized organic matrix (DOM) in acidic or neutral Ph. ^7^

When present in larger quantities, collagen fibrils make the diffusion of acids more difficult during the erosion process. ^8^ Thus, the use of enzymatic inhibitors, especially for MMPs, could reduce tissue loss during subsequent erosive challenges, since the organic matrix would behave as a protective layer. ^8^ Studies have shown good results of MMPs inhibition with chlorhexidine in reducing the degradation of the collagen matrix. ^9 , 10^ However, natural agents have been increasingly studied by researchers because of their lower toxicity and fewer side effects compared to synthetic products. ^11 - 13^

The use of collagen crosslinkers has been successful, showing that 1% grape seed can inactivate 69% of MMPs. ^12^ Moreover, there is a polyphenol called proanthocyanidin (PA), which is easily found in natural products such as grape, cocoa, blackberry and peanut ^12^ and studies have shown that it had the capacity to inactivate more than 90% of MMPs-2, -8 and -9. ^13^ It was demonstrated a significant improvement in adhesion at the resin-dentin attachment interface, making the adhesive restorations resistant and durable. ^11 , 14^ Studies also showed that collagenases had no effect on dentin treated with PA agents, suggesting its inhibitory effect on enzymes that degrade the dentin matrix ^11^ and has the capability to reduce collagen degradation, inhibit demineralization and enhance remineralization in combination with tri-calcium phosphate and fluoride. ^15^

Proanthocyanidin also has showed its effectiveness in preventing dentin erosion, since it could reduce the dentin wear and DOM degradation when used as gel. ^16^

Although green tea and olive oil mouthrinses have been tested and shown good results in the prevention of initial enamel erosion, ^17^ a PA mouthrinse has not been tested yet in dentin erosion. Despite the PA protective effect on collagen degradation, fruits rich in PA such as grape and cranberry have low pH values, ^18^ which accelerate collagen degradation. However, we are not sure whether the naturally acidic PA extracts inhibit or induce demineralized dentin wear.

Many other studies explored the action of PA extracts incorporated in gels for the prevention of dentin erosion. ^16 , 19 , 20 , 21^ However, for easily incorporating this active principles on the patient’s routine, this *in situ/ex vivo* study selected the use of PA extracts as a mouthrinse because it allows greater comfort to the patient, since they can use the solution daily without the need to attend the dental office.

MMPs, although activated, cannot degrade the organic matrix of dentin in acid pH. Therefore, erosive challenges were conducted three time per day, to enhance the degradation of MMP matrix. ^22^ When dentin is exposed to low pH values, demineralization of its inorganic portion occurs and, consequently, exposure of collagen fibrils. At this time, MMPs are activated, but they only start to degrade the exposed collagen when the pH is neutralized. ^23^ Based on that, although the literature has already shown that proanthocyanidin can play an important role in this process, minimizing the damage of dentin erosion, it is still unknown if ideally this agent would have an optimal preventive action at its original pH (3.0) or at neutral pH. Therefore, the aim of this study was to evaluate the protective effect against erosion of a PA mouthrinse in its naturally acidic form and with a neutral pH on dentin subjected to erosive challenges. Thus, the null hypothesis of this study is that there is no difference in the protective effect of the groups tested on dentin subjected to erosion.

## Methodology

### Experimental design

**Figure 1:**
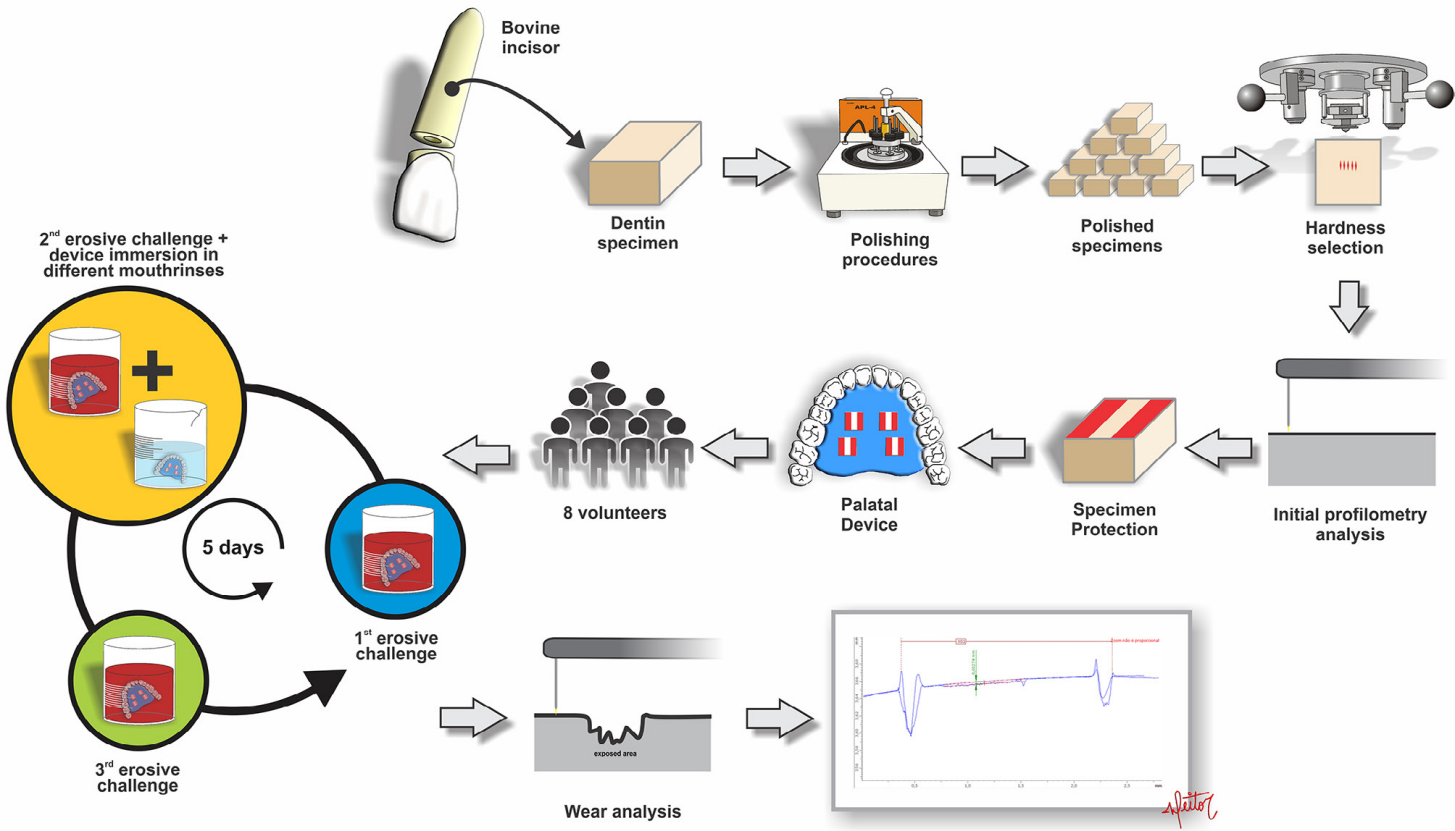
Flowchart of study experimental design

This *in situ* cross-sectional double-blind randomized study was approved by the Research and Ethics Committee of the Bauru School of Dentistry, University of São Paulo (Process: 49813015.7.0000.5417). All the volunteers signed an informed consent form. The study consisted of two phases of five days, with a 7 days washout period between phases. Eight volunteers wore one acrylic palatal device in each phase with 16 samples per group. Each palatal device contained four dentin specimens in a split mouth design (2 specimens per group). Sample size calculation for this study was based on data from a pilot study. Therefore, adopting 0.86 as estimated standard deviation, 0.9 for effect size, 5% as alpha error, 20% for beta error and considering the analysis for 4 dependent groups, a sample size of 12 specimens was estimated. Predicting possible losses, the sample used was 16 specimens for each group in this study. Dentin specimens were divided into 4 groups according to treatment: G1 (test), 10% PA mouthrinse, pH 7.0; G2 (test), 10% PA mouthrinse, pH 3.0; G3 (positive control), 0.12% chlorhexidine mouthrinse, pH 7.0; and G4 (negative control), no treatment. The response variable was dentin wear, measured by profilometry (µm) ( Figure 1 ).

### Dentin specimens' preparation

Each dentin specimen (4×4×3 mm) was obtained from a single root of bovine incisors and was stored in 0.1% thymol solution (pH 7.0) at 4°C. The surfaces of the specimens were polished using water-cooled carborundum discs (Al _2_ O _3_ of 320, 600 and 1200 grits; Buehler, Lake Bluff, IL, USA) and a 1-µm diamond solution (Buehler). Sixty-four specimens were selected based on a mean hardness of 45±7 KHN (Knoop Hardness Number) assessed by five indentations in different regions of the specimen (15 g, 10 s, HMV-2000, Shimadzu Corporation, Tokyo, Japan). The specimens were randomly distributed into 4 groups with 16 samples each, for 8 volunteers.

First, marks were made on the surfaces of the samples using a scalpel for the precise repositioning on the equipment. Using a profilometer (MarSurf GD 25, Gottingen, Germany), five initial surface readings were performed at specific distances: 0.25, 0.5, 0.75, 1.0 and 1.25 μm. The specimens were kept moist and the excess water was carefully removed with filter paper. Afterwards, the marks and the outer two thirds of the surfaces were covered with nail varnish (Cosmed *Indústria de Cosméticos e Medicamento* s, S/A, Barueri, São Paulo, Brazil) to be used as reference surfaces for the final wear analysis. After that, the specimens were sterilized with ethylene oxide and inserted into four cavities (5×5×4 mm), two on each side of a palatal acrylic resin device made specifically for the study. The allocation to the four cavities and the positioning of each group in the palatal device were randomly done.

### Intraoral phase

Eight healthy volunteers (women aged 21-30 years) living in an area with the same tap water fluoride concentration (0.70 mg F/L) participated in this study. The inclusion criteria were normal salivary parameters (stimulated flow rates: 1.88±1.00 mL/min and non-stimulated flow rates: 0.60±0.33 mL/ min), salivary pH of 7.32±0.30, and absence of erosive lesions or untreated caries.

This was a cross-sectional randomized study, so the volunteers were randomized and half of them started at first phase (G1 and G2 groups), whereas the other half started from the second phase (G3 and G4 groups). After a washout period of 7 days, the volunteers changed the phase. Thus, those that started in phase 1 moved to phase 2 and those that started in phase 2 moved to phase 1. At each phase, volunteers wore a new palatal device for five consecutive days. Devices containing the specimens were worn for the first 12 hours of each phase to allow formation of acquired pellicle. ^24^ The erosive challenge was performed *ex vivo* three times a day (8:00 a.m., 2:00 p.m. and 7:00 p.m.). The device was immersed in a cup containing 150 mL of a cola drink (freshly opened bottle) for 5 minutes (Coca-Cola, Companhia Fluminense de Refrigerantes, Porto Real, Rio de Janeiro, Brazil, pH 2.6) at room temperature. After the second erosive challenge of the day, the device was rinsed in running water for 5 seconds, and then one half of the device was immersed in 10% PA mouthrinse pH 7.0 (Purified Grape Seeds Oligomeric Proanthocyanidins, 1298219, Sigma-Aldrich Co. ^®^ , USA) for 5 minutes. It is important to mention that the solution pH was stabilized at 7.0 using a solution of 50% sodium hydroxide. The device was rinsed in running water again for 5 seconds and the other half was immersed in 10% PA mouthrinse pH 3.0 (Purified Grape Seeds Oligomeric Proanthocyanidins, 1298219, Sigma-Aldrich Co. ^®^ , USA) for 5 minutes each time (1 ^st^ phase). In the second phase, only one half of the device was immersed in chlorhexidine solution (positive control group) for 5 minutes, rinsed in running water for 5 seconds and the other half (negative control group) did not receive any treatment – their devices were not immersed in any preventive agent. During the immersion of the palatal device, only half of the appliance was immersed in the indicated solution. For this, a rubber device was created. This device was made using a 6 mm ethyl vinyl acetate (EVA) rubber device, which was cut into a circular shape. In its center, a slit was made, according to the size of the appliance. The palatal acrylic appliance was inserted into the device which should float on the solution, below the rubber. Thus, only half of the acrylic appliance was exposed to the solution, whereas the other half of the appliance was protected from exposure to the solution used in that moment. ^25^ The different mouthrinses used in this study were ordered and handled in a pharmacy (Bauru Fórmulas Farmácia de Manipulação LTDA – ME, SP – Brazil). Therefore, every care was taken to avoid cross-action by agents between groups in *ex vivo* procedures.

The volunteers were instructed to use the devices continuously, including during sleep, and only remove it during the meals (1h, 3 times a day) when the device was stored in moist gauze. Oral hygiene was performed with fluoride-free toothpaste (3% natrosol, 10% glycerin, 2% sodium lauryl sulfate, 0.005% saccharin, 0.1% nipagin, 40% calcium carbonate, 1% menthol – Bauru Fórmulas Farmácia de Manipulação LTDA – ME, SP – Brazil).

### Wear analysis

After the five days of experiment, the nail varnish was removed and the profilometry performed on the same locations as the initial measurements. The dentin samples were kept in 100% humidity until the final analysis to avoid collapsing of the collagen fibrils; the excess water was removed with filter paper before measurement. Dentin loss was estimated by the mean depth of the eroded surface compared to the initial profile using a specific software (MarSurf XCR 20, Gottingen, Germany).

### Statistical analysis

Statistical analysis was performed with SigmaPlot 12.0 (Systat Software Inc. San Jose, CA, USA). Data (n=8 subjects) were checked for normal distribution (Shapiro-Wilk Normality Test) and variance homogeneity (Levene’s Equal Variance Test), and analyzed with repeated ANOVA measures followed by LSD Fisher’s test (p<0.05).

## Results

Table 1 shows the mean wear data. Repeated Measures of Analysis of Variance showed significant differences among tested groups (p=0.004). Fisher *post hoc* test showed no significant difference between G1 and G3 (p=0.919). However, these groups had less dentin wear compared to groups G2 and G4, with no significant differences between them (p=0.182).

**Table 1:** Mean (±SD) of dentine wear (μm) of the study groups (n=8 subjects)

GROUP	WEAR (mean ± SD)
G1 – 10% PA mouthrinse (pH 7.0)	1.17 ^a^ ±0.69
G2 – 10% PA mouthrinse (pH 3.0)	2.99 ^b^ ±1.15
G3 – 0.12% chlorhexidine mouthrinse (pH 7.0)	1.22 ^a^ ±0.25
G4 – No treatment	2.29 ^b^ ±1.13

*Mean values followed by distinct letters presented a statistically significant difference between the groups (repeated measures of ANOVA and LSD Fisher’s Test, p<0.05).

## Discussion

Several agents have been proven to inhibit collagenolytic enzymes in DOM, ^13 , 16 , 19 , 20 , 21 , 23^ with chlorhexidine being the most used solution. ^23 , 26 , 27^ In this study, a mouthwash based on purified PA from grape seed extract was tested because previous studies have demonstrated the PA protective effect on dentin erosion, but using another vehicle. ^16 , 19^

The mouthrinse was selected for the incorporation of PA because it can be used at home. Moreover, studies have demonstrated the efficacy of mouthrinses for different agents in the prevention of initial enamel erosion ^28^ and in reducing dentin erosion and abrasion. ^25^

PA concentration and time-dependent process can cause a brownish coloration of dentin. Reported discolorations are likely related to the content of polymeric, high-molecular weight PA and their oxidation products. Specific information about the chemical species that are colored is unavailable in the literature and some studies are needed to elucidate the underlying chemical mechanisms and determine methods to produce PA materials that do not cause discoloration. ^13^

Chlorhexidine was used as a positive control due to its reported efficacy in the inhibition of collagen degradation by MMPs and ability to decrease dentin wear. ^3 , 6 , 9 , 23 , 25 - 27^ In this study, the groups treated with chlorhexidine and neutralized PA had the best results, without statistical difference. Thus, since a neutral PA-based mouthrinse does not have the side effects of a chlorhexidine mouthrinse, such as tooth staining, loss of taste, and mucosal ulcer, ^29^ its use might be more advantageous for dentin erosion prevention _._

Moreover, PA can present other important features, since it is considered a crosslinking agent. ^13^ Collagen crosslinking agents were tested and the highest inhibition of total MMP activity was observed using grape seed extract, which is rich in PA. ^12^ Although it did not present a significant difference when compared with synthetic agents, one study showed that the grape seed extract provided greater inactivation of MMPs, confirming the better action of natural agents when compared with the synthetic ones. ^12^ In addition, PA is considered a biocompatible agent towards dentin and is available as a renewable resource. ^13^

Besides inhibition of MMPs and induction of crosslinks, which improve dentin mechanical properties, ^11 , 12 , 13 , 30 - 32^ PA also reduces dentin demineralization and increases its remineralization. ^33^ This was showed in artificial root caries lesions, and the mechanism is different than that of fluoride, involving the formation of insoluble complexes that are maintained at acidic pH ^34^ and further bind to the Ca ^2^ ions in saliva, thus increasing remineralization. ^33^

Fruits rich in proanthocyanidin such as grape and cranberry have low pH values ^35^ and, despite the agents that can minimize the collagen degradation exposed to erosive agents, ^11 , 19^ the low pH of their extract itself and/or other organic acids present in the drinks ^35^ may accelerate this process. Therefore, acid PA mouthrinse was unable to prevent dentin wear, since the erosive potential in short acidic challenges, as in this study, is mainly determined by pH. ^36^ In fact, the low pH of the PA mouthrinse may have activated MMPs ^4 , 6^ causing greater degradation of the collagen matrix and, consequently, greater wear. Thus, due to the very low pH in group 2, even considering that PA plays an inhibitory role on MMPs, ^16 , 19^ this agent was could not reduce the erosive process.

Fluoride toothpaste was not part of this study as it has the ability to promote the formation of a metal-rich resistant acid layer or a layer of calcium fluoride that provides temporary protection against erosive challenges. Besides that, other studies have shown the ability of fluoride to reduce collagen degradation ^38^ and inhibit MMPs. ^21^ Thus, in an attempt to isolate the effect of the agents tested in this research, we opted to use a fluoride-free toothpaste.

Profilometry alone on eroded dentin does not reflect mineral loss, since there is interference of collagen matrix. ^39^ Furtheremore, contact profilometers use a diamond-tip stylus moved across the surface to record the surface profile, which is simple, but this traditional method has the potential risk of affecting the reading or even damaging the sample as a consequence of the contact. ^39^ However, despite its limited analysis, this method showed a very strong correlation when compared with a non-contact profilometer and a confocal laser scanning microscope with the same conclusions being separately drawn from data generated on each instrument. ^40^ Previous studies have evaluated dentin loss by comparing its wear. ^16 , 19^ There were no significant difference between PA 10% pH 7.0 and 0.12% chlorhexidine mouthrinses groups in this study, showing less dentin wear compared to the other groups. Previous studies, showed that Proanthocyanidin presented dentin wear reduction with reduced DOM degradation when used in gel. ^16^ Therefore, our findings, made with mouthrinse, only allow us to understand that there was also a reduction in DOM degradation by profilometry, however, studies using addtional tests are needed to confirm this specific purpose.

There were many immersion possibilities to randomize the groups among different treatments in both phases. However, the protocol adopted in this study was immersion of G1 and G3 first in each phase to create a pattern. Due to this fact, the results could be influenced, showing significant lower dentine wear for G1 and G3 when compared with G2 and G4. This could be considered a study limitation.

Another limitation is that it is difficult to extrapolate these results to a clinical situation. This study aimed to simulate the same conditions in all groups to allow a comparison among proposed therapies and to see which would be more efficient in preventing erosive tooth wear. Treatments with different mouthrinses were applied once a day for 5 minutes to simulate a clinically possible condition of daily use of a mouthwash. However, just a randomized clinical trial will present the real clinical effect of these agents. Therefore, based on these preliminary results, the next step should be to clinically evaluate the proanthocyanidin mouthwash to prevent dental erosion.

The null hypothesis was rejected, since this study confirms previous findings that PA reduces dentin wear, ^16 , 19^ emphasizing the importance of pH neutralization, since the neutral PA mouthrinse resulted in lower dentin loss than the acidic PA mouthrinse.

Despite the protective effect for dentin wear, further studies should be conducted to verify if PA has other potential benefits when used as a mouthrinse and confirm the best concentration of the agent.

## Conclusion

The results of this study showed less dentin wear in the 10% PA mouthrinse pH 7.0 and 0.12% chlorhexidine groups, without significant difference. Therefore, we suggest that 10% PA mouthrinse pH 7.0 could be a good strategy to reduce dentin wear progression.
